# Single-Dose Cisplatin Pre-Treatment Enhances Efficacy of ROBO1-Targeted Radioimmunotherapy

**DOI:** 10.3390/ijms21207728

**Published:** 2020-10-19

**Authors:** Kentaro Fujiwara, Keitaro Koyama, Atsushi B. Tsuji, Hiroko Iwanari, Osamu Kusano-Arai, Tatsuya Higashi, Toshimitsu Momose, Takao Hamakubo

**Affiliations:** 1 National Institute of Radiological Sciences, National Institutes for Quantum and Radiological Science and Technology (QST-NIRS), Chiba 263-8555, Japan; ktfuji-tky@umin.ac.jp (K.F.); tsuji.atsushi@qst.go.jp (A.B.T.); higashi.tatsuya@qst.go.jp (T.H.); 2 Department of Radiology, Faculty of Medicine, International University of Health and Welfare, Chiba 286-8686, Japan; keitaro-koyama@iuhw.ac.jp (K.K.); tmomose@iuhw.ac.jp (T.M.); 3 Department of Quantitative Biology and Medicine, Research Center for Advanced Science and Technology, The University of Tokyo, Tokyo 153-8904, Japan; hiwanari@tokumen.co.jp (H.I.); wossam160@yahoo.ne.jp (O.K.-A.); 4 Department of Protein-protein Interaction Research, Institute for Advanced Medical Sciences, Nippon Medical School, Kanagawa 211-8533, Japan

**Keywords:** radioimmunotherapy, cisplatin, combination therapy, small-cell lung cancer, ROBO1

## Abstract

We previously reported that radioimmunotherapy (RIT) using ^90^Y-labeled anti-ROBO1 IgG (^90^Y-B5209B) achieved significant anti-tumor effects against small-cell lung cancer (SCLC) xenografts. However, subsequent tumor regrowth suggested the necessity for more effective therapy. Here, we evaluated the efficacy of combination ^90^Y-B5209B and cisplatin therapy in NCI-H69 SCLC xenograft mice. Mice were divided into four therapeutic groups: saline, cisplatin only, RIT only, or combination therapy. Either saline or cisplatin was administered by injection one day prior to the administration of either saline or ^90^Y-B5209B. Tumor volume, body weight, and blood cell counts were monitored. The pathological analysis was performed on day seven post injection of ^90^Y-B5209B. The survival duration of the combination therapy group was significantly longer than that of the group treated with RIT alone. No significant survival benefit was observed following the isolated administration of cisplatin (relative to saline). Pathological changes following combination therapy were more significant than those following the isolated administration of RIT. Although combination therapy was associated with an increase of several adverse effects such as weight loss and pancytopenia, these were transient. Thus, cisplatin pre-treatment can potentially enhance the efficacy of ^90^Y-B5209B, making it a promising therapeutic strategy for SCLC.

## 1. Introduction

Lung cancer is the most commonly diagnosed cancer and the leading cause of cancer-related deaths worldwide, with small-cell lung cancer (SCLC) accounting for 15% of all lung cancer cases [1,2]. A two-stage classification scheme, such as limited-stage and extensive-stage disease, is routinely used for SCLC clinical staging [3]. Unfortunately, 60–70% of SCLC patients exhibit hematogenous metastases at initial diagnosis [3,4]. Although SCLC is highly sensitive to initial chemotherapy and radiotherapy (methods commonly used to treat SCLC) [5], overall SCLC patient prognosis remains poor. The 2-year survival rate of the limited-stage disease patients is only 20–40%, whereas that of the extensive-stage disease (typically treated with chemotherapy alone) drops precipitously to 5% [3]. Therefore, the development of a combination chemotherapy-radiotherapy regimen applicable to extensive-stage disease is imperative.

Radioimmunotherapy (RIT) is a type of internal radiation therapy applicable to extensive-stage SCLC, as RIT agents can deliver cytotoxic radionuclide to cancer cells, including metastatic cancer cells throughout the patient’s body [6,7]. We previously reported a ROBO1-targeted RIT preclinical study in murine SCLC models [8]. The human ROBO1 gene is a homolog of the *Drosophila* Roundabout gene and acts as a cell-surface receptor for slit2 [9,10]. Such slit2–ROBO1 interactions mediate repulsive cues in axons and growth cones during neural development [9,10]. In cancer, ROBO1 contributes to both metastasis and angiogenesis [11,12,13]. Moreover, it is frequently expressed in hepatocellular carcinoma and small-cell lung cancer [14,15]. We previously developed an RIT agent—^90^Y-labeled anti-ROBO1 IgG B5209B (^90^Y-B5209B)—and conducted studies in ROBO1-positive SCLC xenograft mice [8]. Although ^90^Y-B5209B induced anti-tumor effects (e.g., tumor cell death and shrinkage), tumor regrowth after day 21 post injection highlighted the need to improve the efficacy of therapy to achieve complete remission [8].

Cisplatin is a chemotherapeutic drug used to treat lung cancer, including SCLC [5,16]. A combination of radiotherapy and platinum-based chemotherapy is generally used for SCLC treatment, although this approach is restricted to limited-stage disease since external-beam radiation therapy is unsuitable for extensive-stage disease [5]. Unlike external-beam radiation therapy, RIT can target metastatic foci, and therefore has the potential for combination with chemotherapy in the treatment of extensive-stage SCLC. In addition, cisplatin is also a radiosensitizer [17,18] with the potential to enhance the anti-tumor effect of ^90^Y-B5209B.

The present study evaluated the impact of a combination of cisplatin pre-treatment and ^90^Y-B5209B-based RIT in a murine SCLC xenograft model. Specifically, the survival benefit, histopathologic tumor response, and adverse effects were investigated.

## 2. Results

### 2.1. In Vivo Therapeutic Study

**Figure 1:**
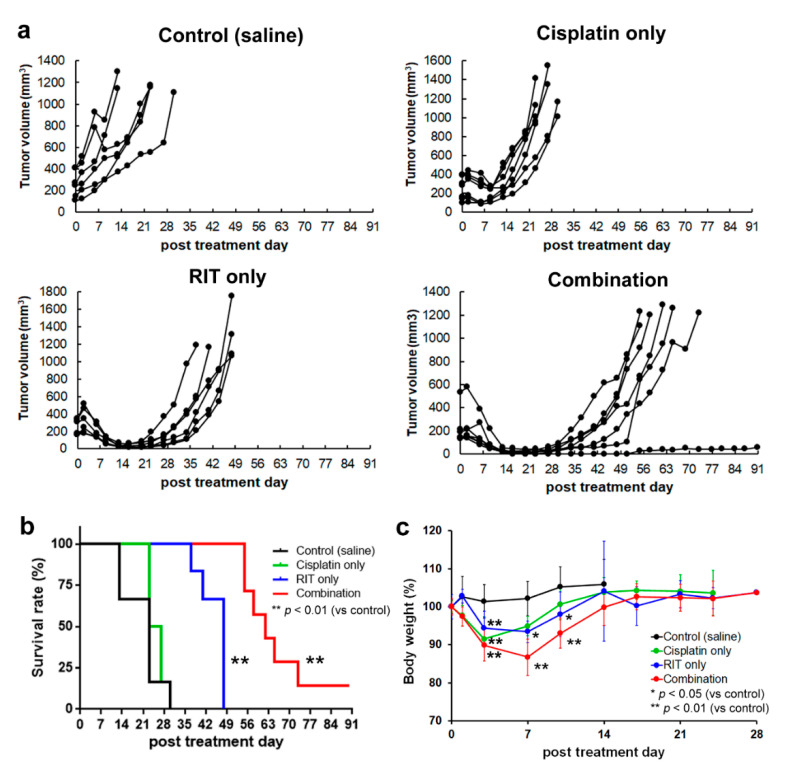
Therapeutic impact and adverse effects observed in distinct treatment groups. (**a**) Change in tumor volume of the control group; (**b**) Kaplan–Meier survival curves; (**c**) Change in body weight. Black represents the control group, green represents the cisplatin-only group, blue represents the radioimmunotherapy (RIT)-only group, and red represents the combination therapy group. * *p* < 0.05 (vs. control group), ** *p* < 0.01 (vs. control group).

The results are illustrated in Figure 1. All intervals are reported as days following treatment. Regarding tumor volume (Figure 1a), baseline measurements were as follows: 223.8 ± 104.0 mm^3^ (control group, i.e., receiving only saline), 222.6 ± 102.8 mm^3^ (cisplatin-only group), 240.0 ± 110.4 mm^3^ (RIT-only group), and 221.5 ± 140.1 mm^3^ (combination therapy group). All mice in the control group exhibited tumor enlargement, whereas the other groups exhibited tumor regression to 87.9 ± 20.7% (cisplatin-only; transient effect lasting nine days), 12.1 ± 6.2% (^90^Y-B5209B-based RIT-only), and 8.0 ± 8.4% (combination therapy). Thus, tumor volume decreased to a progressively greater extent and for a longer duration with increasing treatment aggressiveness. The median survival time (MST) in the control group was 27 days, whereas the other groups exhibited MSTs of 30 days (cisplatin-only), 51 days (RIT-only), and 65 days (combination therapy) (Figure 1b). Thus, survival improved progressively with increasing treatment aggressiveness. The difference in MST between the control and cisplatin-only groups was not significant, whereas combination therapy significantly extended MST relative to each of the other groups (*p* < 0.01). A single mouse in the combination therapy group survived 90 days (the endpoint of the observation period). Regarding body weight (Figure 1c), the control group exhibited no significant weight loss, whereas the other groups exhibited a transient weight loss to 91.5 ± 2.7% (cisplatin-only), 90.4 ± 8.8% (RIT-only), and 86.4 ± 4.0% (combination therapy). Thus, weight loss increased progressively with increasing treatment aggressiveness. The difference in weight loss between groups receiving only cisplatin versus combination therapy was significant only on days seven and ten. The difference in weight loss between groups receiving only RIT versus combination therapy was non-significant at all time-points.

**Figure 2:**
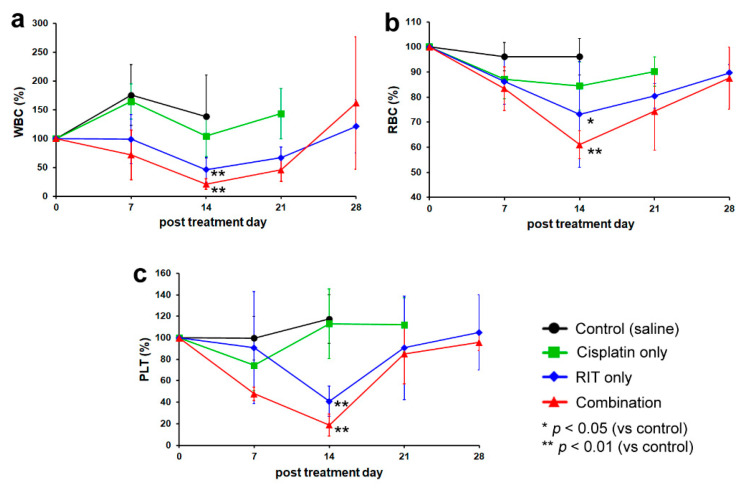
Peripheral blood count trends across distinct treatment groups. (**a**) Change in white blood cell (WBC) count; (**b**) Change in red blood cell (RBC) count; (**c**) Change in platelet (PLT) count. Black represents the control group, green represents the cisplatin-only group, blue represents the RIT-only group, and red represents the combination treatment group. * *p* < 0.05 (vs. control group), ** *p* < 0.01 (vs. control group).

Peripheral blood counts—including white blood cell (WBC) count, red blood cell (RBC) count, and platelet (PLT) count—were performed daily for 28 days following treatment (Figure 2). Neither the control nor the cisplatin-only groups exhibited a significant decrease in peripheral blood counts. No significant differences in peripheral blood counts were observed between these groups at any time point. However, both the RIT-only and combination therapy groups exhibited transient pancytopenia. In the RIT-only group, WBC, RBC, and PLT counts decreased to 41.0 ± 24.1%, 70.8 ± 20.2%, and 40.2 ± 12.9%, respectively. In the combination therapy group, WBC, RBC, and PLT counts decreased to 20.3 ± 8.6%, 61.6 ± 5.3%, and 19.0 ± 9.6%, respectively. Thus, peripheral blood counts decreased progressively with increasing treatment aggressiveness. Differences between the control or cisplatin-only groups versus the RIT-only or combination treatment groups were significant. Although counts were lower in the combination treatment than the RIT-only group, differences were non-significant.

### 2.2. Ex Vivo Tumor and Organ Histopathology

**Figure 3:**
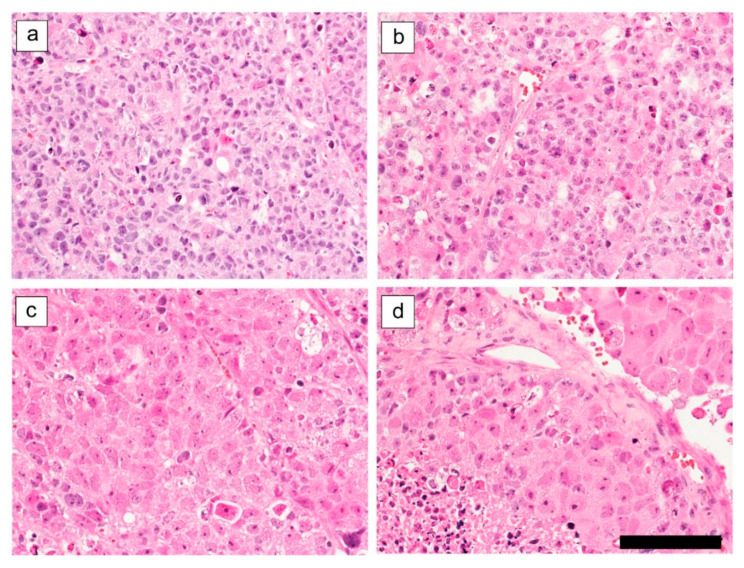
Tumor specimen histology across distinct treatment groups (400× magnification, scale bar: 100 µm). (**a**) Control group; (**b**) Cisplatin-only group; (**c**) RIT-only group; (**d**) Combination therapy group.

Representative day seven tumor specimen histology is shown in Figure 3. Control group tumor tissue demonstrated solid growth within hyalinized fibrous stroma (Figure 3a). Tumor tissue in the cisplatin-only group demonstrated evidence of mild cell denaturation, such as cell body swelling (Figure 3b). Tumor tissue of the RIT-only group demonstrated massive coagulative necrosis and evidence of cell denaturation, including cell body swelling and chromatin condensation (Figure 3c). Tumor tissue in the combination treatment group demonstrated more extensive massive coagulative necrosis and evidence of cell denaturation, including cell body swelling and chromatin condensation (Figure 3d).

**Figure 4:**
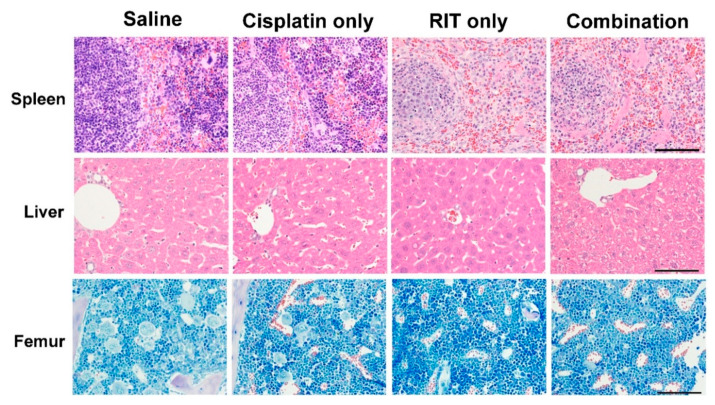
Day 7 organ specimen histopathology (400× magnification, scale bar: 100 μm).

Representative day seven organ specimen histology is shown in Figure 4. No apparent pathological changes were evident in organ tissues of the control or cisplatin-only groups. Only splenic tissue of the RIT-only group demonstrated a remarkable decrease in red pulp hematopoietic cells and white pulp lymphocytes; changes were also observed in the combination treatment group. Only the combination treatment group demonstrated decreased femur hematopoietic cells (especially megakaryocytes) and hepatocyte ballooning. Thus, organ pathology increased progressively with increasing treatment aggressiveness.

**Figure 5:**
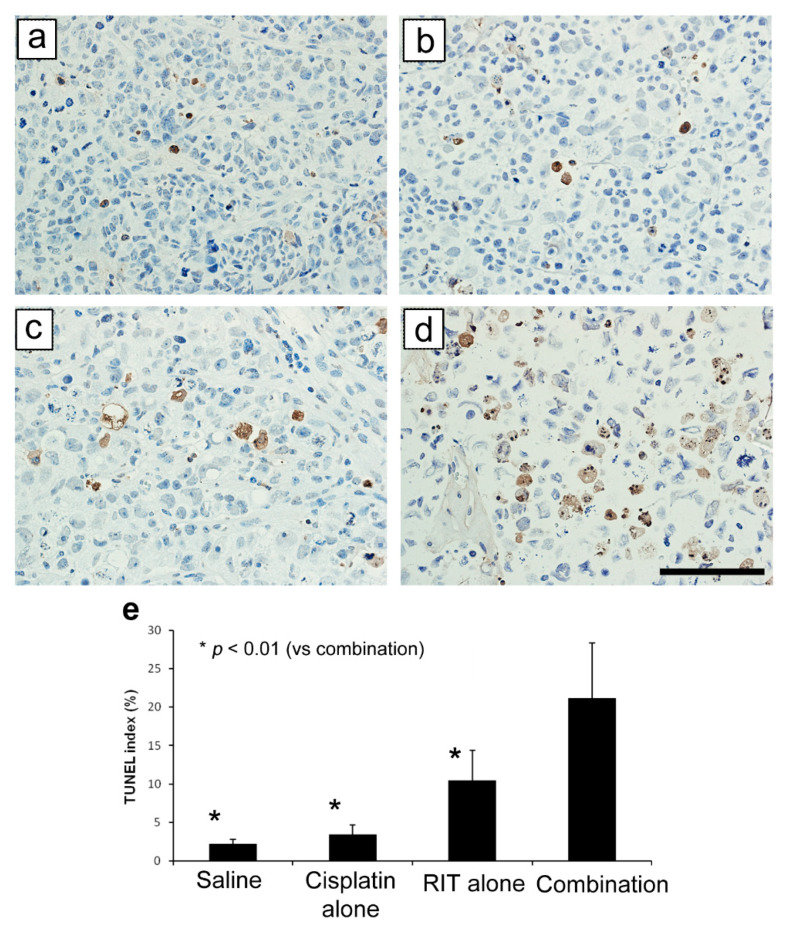
Day seven TUNEL analysis across distinct treatment groups (400× magnification, scale bar: 100 µm). (**a**) Control, (**b**) Cisplatin-only, (**c**) RIT-only, and (**d**) Combination therapy groups with associated TUNEL indices (**e**). * *p* < 0.01 (vs. combination group).

Terminal deoxynucleotidyl transferase dUTP nick end labeling (TUNEL) indices are shown in Figure 5: 2.2 ± 0.6% (control group), 3.4 ± 1.3% (cisplatin-only group), 10.5 ± 3.9% (RIT-only group), and 21.1 ± 7.2% (combination treatment group). Thus, indices increased progressively with increasing treatment aggressiveness. The index difference between the control and cisplatin-only groups was non-significant. However, the RIT-only group index was significantly higher than that of the control and the cisplatin-only groups (*p* < 0.05), and the combination group index was significantly higher than that of any of the other three groups (*p* < 0.01).

## 3. Discussion

The present study evaluated the impact of a combination of cisplatin pre-treatment and ^90^Y-B5209B-based RIT in a murine SCLC xenograft model, and demonstrated that cisplatin pre-treatment significantly enhanced RIT efficacy (while also worsening transient weight loss and pancytopenia). Although cisplatin alone transiently suppressed tumor growth, it offered no survival benefit, while RIT alone and in combination with cisplatin pre-treatment significantly prolonged survival. Combination therapy offered a significant benefit over RIT alone by prolonging survival by a median of 14 additional days. Histopathology demonstrated results consistent with those of the therapeutic study. Collectively, these results suggest that cisplatin pre-treatment enhances the anti-tumor effects of ^90^Y-B5209B-based RIT while also enhancing adverse effects, albeit transiently.

Cisplatin likely enhances the anti-tumor effects of ^90^Y-B5209B via the radiosensitizing effects of platinum drugs [18]. Although incompletely understood, two major hypotheses exist regarding the mechanism of radiosensitization. One involves DNA platination. Platinum drugs bind DNA to form Pt-DNA lesions, which may suppress non-homologous end-joining-mediated DNA repair, thereby inhibiting proliferation or inducing cell death when Pt-DNA lesions are proximal to double-strand breaks generated by ionizing radiation [19,20]. The other hypothesis involves the induction of cell-cycle arrest [18]. Cisplatin DNA binding can induce G1 and G2/M arrest, thereby inhibiting replication (the latter synchronizing cells in the G2/M phase in a dose-dependent manner until several days after in vitro treatment) [21,22]. It is well known that cells in the G2/M phase exhibit enhanced radiosensitivity (relative to cells in other phases of the cell cycle) [23]. Thus, cisplatin-exposed cells are likely to remain radiosensitive for several days post exposure. In the present study, cisplatin treatment preceded ^90^Y-B5209B administration. Tumor ^90^Y-B5209B uptake has been reported to plateau at two to three days following administration [8,24]. Therefore, when combined with cisplatin pre-treatment, this plateau would coincide with tumor cell G2/M phase synchronization, and therefore would enhance radiosensitivity. This may explain the observations of the present study indicating that single-dose cisplatin pre-treatment enhances RIT efficacy.

While combination treatment amplified adverse effects (weight loss and pancytopenia) relative to RIT alone, these were well-tolerated and transient: body weight and peripheral blood counts returned to their original values by the end of the observation period. Histopathology demonstrated changes in hematopoietic tissue (spleen and femur), consistent with the above results. Since hematopoietic tissues exhibit high proliferative rates (and thus radiosensitivity), it is unsurprising that they were impacted to a greater extent than somatic tissues. However, cisplatin administration is systemic (via circulation) and therefore enhances RIT effects not only on tumor cells but also on normal tissues. For translation to the clinic, the side effects of the combination therapy should be managed, while it was well-tolerated in the treatment schedule in mice. Cisplatin nanoparticles could be promising to decrease the side effects because of the sustained release of cisplatin from the particles [25]. In the clinical setting, combination therapy would require careful consideration of the implications of these adverse effects, as well as their monitoring during treatment.

Although combining cisplatin pre-treatment with ^90^Y-B5209B-based RIT demonstrated enhanced anti-tumor effects and prolonged survival in SCLC xenograft mice, it did not achieve SCLC cure. Several potential curative strategies include the following. First, optimization of combination treatment dosage and timing would maximize SCLC xenograft radiosensitivity and the therapeutic effect. While a cisplatin (radiosensitizer) dose of 5 mg/kg enhanced ^90^Y-B5209B efficacy, the anti-tumor effect of cisplatin was limited. Dose escalation and continuous administration of cisplatin may improve the therapeutic effect. Second, combining an antibody–drug conjugate (ADC) with RIT may be a promising strategy to enhance therapeutic effects without a concomitant increase in adverse effects. The superior tumor specificity and potency of ADCs relative to traditional drugs may decrease non-specific uptake by organs not afflicted by tumor [26]. Indeed, Sharkey et al. reported the feasibility of combining ADC and RIT for improved efficacy without increased toxicity [27]; ADC combined with ^90^Y-B5209B-based RIT may enhance anti-tumor effects without enhancing adverse effects. Third, radionuclides with superior cytotoxicity, such as ^225^Ac, have the potential to exert enhanced anti-tumor effects (relative to ^90^Y). Specifically, ^225^Ac is a nanogenerator nuclide, generating daughter radionuclides with shorter 10-day half-lives, each emitting four high-energy α particles per decay cycle [28]. Kratochwil et al. reported a prostate-specific membrane antigen (PSMA)-targeted α-radiation therapy (^225^Ac-PSMA-617) which induced complete remission in prostate cancer patients (including decreasing PSA levels to less than 0.1 ng/mL) without hematologic toxicity [29]. Such results strongly suggest the potential for significant benefits of this therapy in advanced-stage prostate cancer patients. Thus, ^225^Ac-labeled B5209B may have a superior therapeutic effect (relative to ^90^Y-B5209B), and may prove curative in combination with chemotherapeutic agents.

In conclusion, cisplatin pre-treatment enhances the efficacy of ^90^Y-B5209-based RIT in a murine SCLC xenograft model. Moreover, the optimization of this combination treatment strategy may minimize adverse effects and prove therapeutic or, indeed, curative in extensive-stage disease.

## 4. Materials and Methods

### 4.1. Cell Cultures and Animal Models

A ROBO1-positive SCLC cell line—NCI-H69 (ATCC HTB-119, Manassas, VA, USA)—was obtained from the American Type Culture Collection and cultured as previously described [8]. Five-week-old male BALB/c nude mice were purchased from Clea Japan, Inc. (Tokyo, Japan), and NCI-H69 xenograft mice were generated as previously described [8]. All animal study protocols were approved by the Animal Care Committee of the University of Tokyo (ethics approval number: P10–017, 28 April 2010).

### 4.2. Antibodies

A murine monoclonal IgG2b antibody against human ROBO1 was raised as previously described [24] and was termed B5209B. It does not cross-react with murine ROBO1.

### 4.3. Chelate Conjugation and Radiolabeling

Bifunctional chelator: 1,4,7,10-tetraazacyclododecane-1,4,7,10-tetraacetic acid (DOTA) was purchased from Macrocyclics (Dallas, TX, USA), and ^90^YCl3 was obtained from Eckert and Ziegler (Braunschweig, Germany). Chelate conjugation and radiolabeling of B5209B were performed by FUJIFILM Toyama Chemical Co., Ltd. (Chiba, Japan), as previously described [24]. Immunoreactivity of ^90^Y-labelled B5209B (^90^Y-B5209B) is not impaired relative to unlabeled B5209B [24]. Labeling yield (approximately 80%) and radiochemical purity (approximately 99.3%) were estimated via instant thin-layer chromatography (Pall Corp., Port Washington, NY, USA).

### 4.4. Therapeutic Study

**Figure 6:**
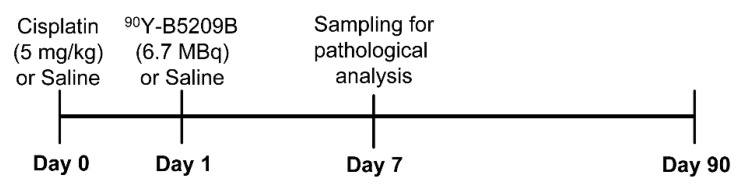
Therapeutic study experimental schedule.

Xenograft mice were randomized to four groups (*n* = 6–7 per group): saline (control), cisplatin-only, RIT-only, and combination (cisplatin and RIT) therapy. Injection doses (per mouse) of cisplatin and ^90^Y-B5209B were 5 mg/kg and 6.7 MBq (80 μg), respectively. On day 0, mice received saline or cisplatin intraperitoneally (Figure 6). On day 1, mice received saline or ^90^Y-B5209B. The present study made use of single-dose cisplatin and ^90^Y-B5209B. Tumor volume and body weight were assessed twice per week until 90 days following treatment. Tumor volume was calculated using the following formula: 0.5 × (shortest diameter)^2^ × (longest diameter). Tumor growth (%) was calculated using the formula: (tumor volume at each time point)/(tumor volume on day 0) × 100. Peripheral blood counts, including white blood cell (WBC) count, red blood cell (RBC) count, and platelet (PLT) count were measured once a week until four weeks following treatment. Peripheral blood was collected from the tail vein and subjected to automated hematology using a Celltac α analyzer (MEK-6400; Nihon Kohden, Tokyo, Japan). Mice were euthanized by isoflurane inhalation once tumor volume reached 1000 mm^3^ or weight loss exceeded 20%.

### 4.5. Pathological Study

Xenograft mice were randomized to four groups (*n* = 2 per group): saline (control), cisplatin alone, RIT alone, and combination (cisplatin and RIT) therapy. Mice were euthanized seven days following RIT treatment, and specimens of NCI-H69 tumor as well as tumor-unaffected tissues (liver, kidney, lung, heart, intestine, spleen, pancreas, and femur) were obtained. All specimens were fixed overnight in 4% paraformaldehyde at 4 °C, embedded in paraffin, and cut into 3 μm sections. Femurs were decalcified prior to paraffin embedding. Tissue sections were stained using hematoxylin and eosin (H&E), excluding femoral bone sections, which were stained using Giemsa solution. To determine apoptotic status, TUNEL analysis was performed on tumor sections. Apoptosis was detected using the ApopTag Plus Peroxidase In Situ Apoptosis Detection Kit (Chemicon International, Inc., Billerica, MA, USA), according to the manufacturer’s instructions. Apoptotic cells were quantified by determining the percentage of TUNEL-positive cells (TUNEL index), as previously described [24].

### 4.6. Statistical Analyses

Statistical analyses were performed with Graphpad Prism software (version 6.07; Graphpad Software, San Diego, CA). Data were expressed as the mean ± standard deviation. Means were compared using a one-way ANOVA and Tukey–Kramer HSD test. Log-rank tests were performed to compare survival outcomes. Results with *p* < 0.05 were considered statistically significant.

## Abbreviations

SCLC	Small-cell lung cancer
RIT	Radioimmunotherapy
MST	Median survival time
WBC	White blood cell
RBC	Red blood cell
PLT	Platelet
DOTA	1,4,7,10-tetraazacyclododecane-1,4,7,10-tetraacetic acid
TUNEL	Transferase-mediated dUTP nick end labeling
ADC	Antibody–drug conjugate
PSMA	Prostate-specific membrane antigen
